# An Inducible Cell-Cell Fusion System with Integrated Ability to Measure the Efficiency and Specificity of HIV-1 Entry Inhibitors

**DOI:** 10.1371/journal.pone.0026731

**Published:** 2011-11-01

**Authors:** Alon Herschhorn, Andres Finzi, David M. Jones, Joel R. Courter, Akihiro Sugawara, Amos B. Smith, Joseph G. Sodroski

**Affiliations:** 1 Department of Immunology Cancer and AIDS, Dana-Farber Cancer Institute and Department of Microbiology and Immunobiology, Harvard Medical School, Boston, Massachusetts, United States of America; 2 Department of Chemistry, University of Pennsylvania, Philadelphia, Pennsylvania, United States of America; 3 Department of Immunology and Infectious Diseases, Harvard School of Public Health, Boston, Massachusetts, United States of America; McGill University AIDS Centre, Canada

## Abstract

HIV-1 envelope glycoproteins (Envs) mediate virus entry by fusing the viral and target cell membranes, a multi-step process that represents an attractive target for inhibition. Entry inhibitors with broad-range activity against diverse isolates of HIV-1 may be extremely useful as lead compounds for the development of therapies or prophylactic microbicides. To facilitate the identification of such inhibitors, we have constructed a cell-cell fusion system capable of simultaneously monitoring inhibition efficiency and specificity. In this system, effector cells stably express a tetracycline-controlled transactivator (tTA) that enables tightly inducible expression of both HIV-1 Env and the Renilla luciferase (R-Luc) reporter protein. Target cells express the HIV-1 receptors, CD4 and CCR5, and carry the firefly luciferase (F-Luc) reporter gene under the control of a tTA-responsive promoter. Thus, Env-mediated fusion of these two cell types allows the tTA to diffuse to the target cell and activate the expression of the F-Luc protein. The efficiency with which an inhibitor blocks cell-cell fusion is measured by a decrease in the F-Luc activity, while the specificity of the inhibitor is evaluated by its effect on the R-Luc activity. The system exhibited a high dynamic range and high Z'-factor values. The assay was validated with a reference panel of inhibitors that target different steps in HIV-1 entry, yielding inhibitory concentrations comparable to published virus inhibition data. Our system is suitable for large-scale screening of chemical libraries and can also be used for detailed characterization of inhibitory and cytotoxic properties of known entry inhibitors.

## Introduction

Human immunodeficiency virus type-1 (HIV-1) is a retrovirus that causes acquired immunodeficiency syndrome (AIDS) in humans. HIV-1 establishes a persistent infection that, in the absence of treatment, results in the severe depletion of CD4-expressing lymphocytes and usually fatal immunodeficiency [1], [2]. Antiretroviral therapy for HIV-1 infection combines inhibitors against several functional proteins of HIV-1, including the viral reverse transcriptase, protease, gp41 and integrase, and also includes a ligand of the CCR5 co-receptor that blocks viral entry [3]. The use of a combination of drugs efficiently decreases virus loads and extends the lifespan of HIV-1-infected individuals. However, despite the large and effective arsenal available to fight HIV-1, resistant variants of HIV-1 eventually evolve during therapy; moreover, some antiretroviral drugs exhibit long-term toxicity [3], [4], [5], [6], [7], [8]. Thus, it is essential to identify additional new inhibitors with low cytotoxicity and broad-range activity against diverse HIV-1 strains for future success in treating HIV-1 infection. In addition to their use as therapeutics, such inhibitors may be also used to prevent HIV-1 transmission. This strategy has been validated in the recent partial success of tenofovir, a reverse transcriptase inhibitor, in preventing sexual transmission of HIV-1 when it was administrated either orally or as a topical microbicide [9], [10].

The HIV-1 envelope glycoproteins (Envs) mediate virus entry into cells, and represent attractive targets for intervention. Three gp120 exterior Envs and three gp41 transmembrane Envs are assembled into the trimeric envelope spike and anchored on the HIV-1 virion surface by the gp41 membrane-spanning segments [11], [12], [13]. The gp120 glycoprotein binds the CD4 receptor and either the CCR5 or CXCR4 chemokine coreceptor [14], [15], [16], [17]. Receptor binding moves the high-potential-energy Env complex into lower-energy forms, culminating in the formation of a six-helix bundle in gp41 that mediates the fusion of the viral and target cell membranes [18], [19], [20]. The high potential energy and level of exposure of the Envs create opportunities for premature, irreversible inactivation by small molecules [21]. In addition, entry inhibitors may also block viral interaction with the host receptors or interfere with critical conformational transitions of the envelope proteins during membrane fusion.

Several inhibitors of the HIV-1 Envs have been developed, targeting different sites either on the Envs or on the co-receptors that are required for virus entry. A few small molecules such as NBD-556 and BMS-378806 (BMS-806) interact with gp120 and prematurely trigger or interfere with conformational changes in the Envs, respectively [22], [23], [24], [25], [26], [27], [28], [29]. Small-molecule ligands of CCR5 and CXCR4 bind to the cognate coreceptor leading, in most cases, to a conformation that is not recognized by HIV-1 Env, and less frequently, to coreceptor internalization [30], [31], [32], [33], [34], [35], [36]. The gp41-derived peptide T20 competes with the gp41 heptad repeat 2 (HR2) region in the intact Envs on virions and blocks subsequent steps required for membrane fusion [37]. Several of these inhibitors are very potent and two of them (T20 and Maraviroc, a CCR5 ligand) have been approved for treatment of HIV-1 infection. However, under the selective pressure of these molecules, resistant variants of HIV-1 eventually emerge, impeding long-term efficacy of the compounds and underscoring the urgent need for new and broad-range inhibitors [38], [39], [40], [41], [42]. Inhibitors that impose a high genetic barrier to the development of resistance could lead to novel treatment or prophylactic options. Such inhibitors could also serve as extremely useful probes to study the poorly understood transitions of the HIV-1 Envs to different conformational intermediates along the virus entry pathway.

The ability of the HIV-1 Envs to mediate membrane fusion at neutral pH results in Env-expressing cells fusing with receptor-expressing cells to form multinucleated syncytia [43], [44]. Cell-cell fusion assays are attractive tools for identifying potent inhibitors of HIV-1 Env function. Such assays mimic the entry steps of HIV-1 into cells, and can measure membrane-fusing activity, as well as its inhibition, with high sensitivity [43], [44], [45], [46], [47], [48], [49], [50], [51], [52]. The assay also simulates the multiple Env-receptor interactions that occur during direct transmission of the virus from one cell to another, and offers a unique system to study and potentially inhibit this mode of transmission [53], [54], [55]. Cell-cell fusion systems are typically composed of two types of cells: effector cells that express the HIV-1 Envs and target cells that express the CD4 receptor and CCR5 (or CXCR4) coreceptor. During cocultivation, HIV-1 Envs expressed on the surface of effector cells bind receptor-bearing target cells, and fuse the membranes of the cells, leading to the formation of multinucleated syncytia [43], [44]. The fusion event can be readily detected by inducing expression of a reporter protein in the target cells. Two different cell-cell fusion assays have been independently developed for large-scale screening [56], [57]. Although they are effective and robust, both can benefit from further improvement in their design and use. The first system utilized effector cells that stably express Envs on the surface of Chinese hamster ovary (CHO) cells; however, because long-term Env expression may exert subtle cytotoxic effects, the proportion of correctly folded and processed Envs on the surface of such effector cells may diminish with time [56]. An intensive screen with this system has successfully identified the potent inhibitor PF-68742; however, a single residue change, Gly514Arg (standard HXB2 numbering) in gp41, confers resistance to the compound [58]. The second assay was configured as two systems, one for screening CCR5-mediated entry inhibitors and the other for screening CXCR4-mediated entry inhibitors [57]. Screening a library of compounds with the CCR5-mediated system and excluding any compounds that also decreased the CXCR4-mediated fusion identified CCR5-specific inhibitors. While effective, this strategy would clearly fail to identify compounds with broad-range activity that would be expected to inhibit both CCR5 and CXCR4-mediated entry. Here we present a cell-cell fusion system that applies a different strategy and carries a built-in capability to measure non-specific inhibition; the system incorporates the most advanced elements in a cell-cell fusion system. Each protein is expressed from a codon-optimized gene, and response elements are tightly regulated and have low background activity. The system is robust, has high dynamic range, and can sequentially measure the inhibition potency and specificity of each compound in the same cells.

## Results

### Design of cell-cell fusion system

**Figure 1:**
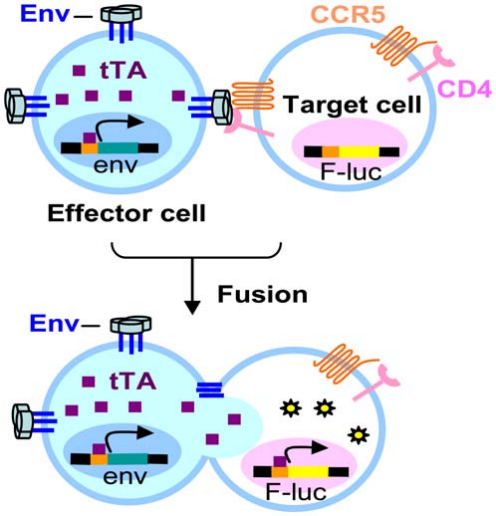
Assay scheme for HIV-1 Env-mediated cell-cell fusion. An activator (tTA) is used for inducible expression of the HIV-1 Envs in the effector cells, allowing Env expression to be turned on by the removal of Dox from the medium. When effector and target cells are cocultivated, cell-cell fusion enables the diffusion of tTA to the target cell. This activates the transcription of the F-Luc reporter gene, which is also under the control of a tTA responsive promoter. F-Luc activity can be measured and is quantitatively related to the extent of fusion.

We have designed and built a unique cell-cell fusion system that incorporates several features optimized for the study of HIV-1 Env-mediated membrane fusion and its inhibition. Cell-cell fusion occurs when effector cells expressing HIV-1 Envs are cocultivated with target cells that express the appropriate receptors. For certain applications, constitutive expression of HIV-1 Envs may be important, but Env-related toxicity in cells may counterselect cells that stably express high levels of correctly processed Envs. To avoid such an outcome, we have used an inducible Env expression system that utilizes a tetracycline-controlled transactivator (tTA) protein and is tightly regulated by doxycycline (Dox, a tetracycline analogue) [59], [60]. Effector cells were generated by transferring the codon-optimized HIV-1 *env* gene under the control of a tTA-responsive promoter into HeLa-TetOff cells, which constitutively express optimal levels of tTA (Figure 1). The tTA-mediated transcription of Env can be turned on or off by removing Dox from or adding Dox to the medium, respectively. This biological switch ensures tight control of Env expression, which is induced only immediately prior to the fusion assay, thus avoiding detrimental effects of Envs during propagation of the effector cells.

To minimize the complexity of the system, the Env-inducing tTA protein in the effector cells was also used to activate reporter gene expression in the target cells (Figure 1). For this purpose, the firefly luciferase (F-luc) gene, under the control of a tTA-responsive promoter, was transferred into target cells that express CD4 and CCR5 receptors. Thus, fusion of the effector and target cells enables the tTA to diffuse from the effector cell to the target cell and activate the expression of F-Luc protein. F-Luc activity is proportional to the extent of cell-cell fusion.

### Stable effector and target cells

**Figure 2:**
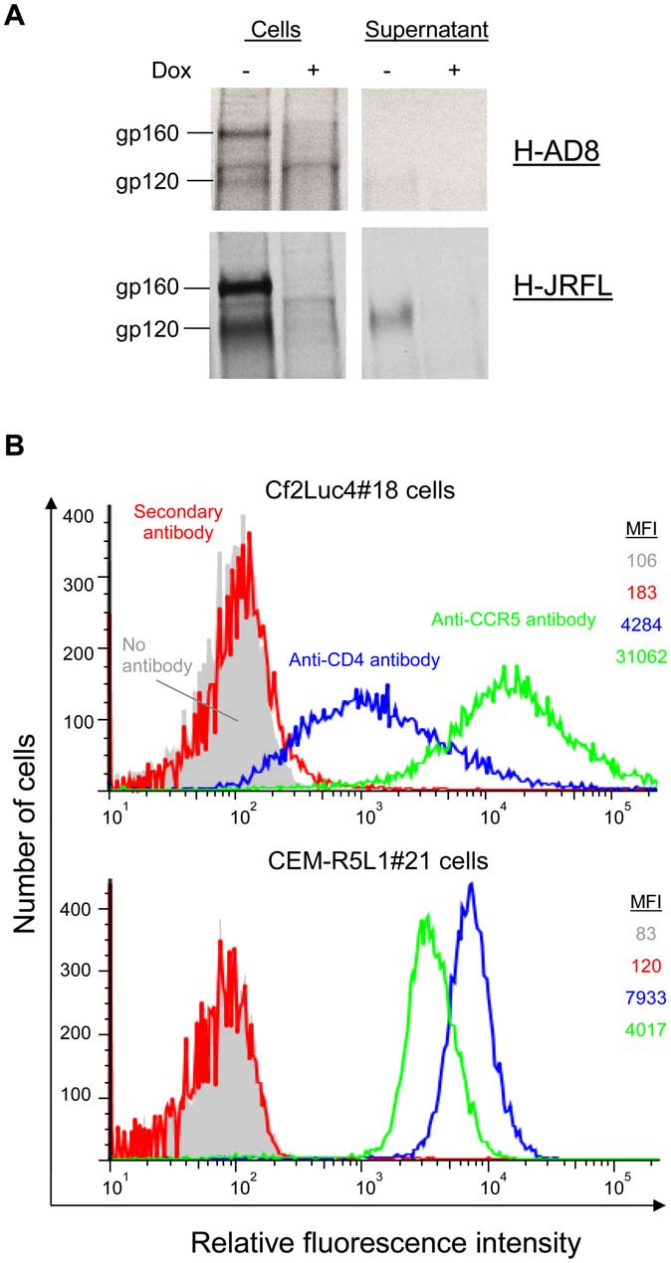
Expression of HIV-1 Envs in effector cells and HIV-1 receptors in stable target cells. **A.** The H-AD8 and H-JRFL cells were induced for 48 hours and then metabolically labeled with ^35^S overnight in the absence of Dox. Control cells were treated similarly but 2 µg/ml Dox was present in the medium at all times. Cell lysates and supernatants were subjected to radioimmunoprecipitation and analysis on polyacrylamide gels. **B**. Levels of expression of CD4 and CCR5 receptors on the two target cells were analyzed by flow cytometry. Cells were stained with secondary antibody alone as a control, or with either OKT4 (anti-CD4) antibody or 2D7 (anti-CCR5) antibody followed by the secondary antibody. Color codes are identical for both target cells. MFI, mean fluorescence intensity.

Three different vectors were tested for the ability to express two different primary HIV-1 Envs transiently in effector cells. Transfected cells were also tested for membrane-fusing activity by cocultivation with target cells (Figure S1 and Method S1). Based on these results, plvx-AD8 and pGL4.2-JRFL, which express the Envs of two primary HIV-1 isolates, AD8 and JR-FL, respectively, were selected. These plasmids were used to generate the stable HeLa-AD8 (H-AD8) and HeLa-JRFL (H-JRFL) cells, respectively. Expression of Envs was detected in both stable effector cells, and was controlled by the presence or absent of Dox in the medium (Figure 2A). Similarly to transient expression (Figure S1), stable Env expression from the pGL4.2 vector resulted in higher Env levels relative to expression from plvx-AD8, and allowed the detection of shed gp120 in the supernatant of the expressing cells.

Two different target cell lines were generated by lentivirus-mediated gene transfer of the F-Luc gene, under the control of the tTA-responsive promoter. Both cells express human CD4 and CCR5. Cf2Luc4#18 cells are canine Cf2Th cells, whereas CEM-R5Luc1#21 cells are human T lymphocytes. Both were derived from single clones and were selected for their low background of fusion with Env-expressor cells in the presence of Dox and high signal with Env-expressing cells (in the absence of Dox). Flow cytometric analysis of CD4 and CCR5 levels on the surface of these cells showed that Cf2Luc4#18 cells express low-to-moderate levels of CD4, but high levels of CCR5. In contrast, CEM-R5Luc1#21 cells express high levels of CD4 and lower levels of CCR5 (Figure 2B). Thus, different combinations of receptor levels are represented on the surface of these two cell lines.

**Figure 3:**
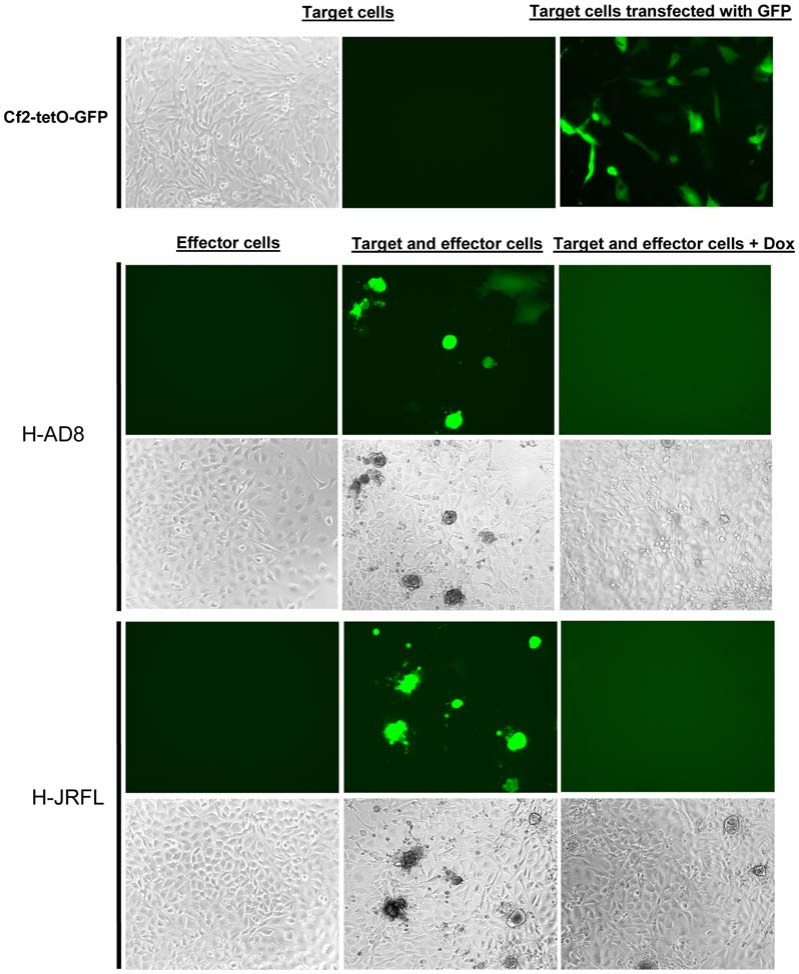
Direct visualization of cell-cell fusion with stably integrated and inducible GFP gene in target cells. The H-AD8 and H-JRFL effector cells were incubated separately with Cf2-tetO-GFP cells; forty hours later, cells were visualized under a fluorescence microscope. Images were acquired for target and effector cells alone and after cocultivation. The phase contrast and fluorescence images are shown for each culture, except for the transiently transfected cells. Cf2Th-CD4/CCR5 cells that were transiently cotransfected with the plvx-tetO-GFP and tTA expression plasmids (plvx-TetOff Advanced, Clontech) were visualized after 24 hours and are shown in the right top panel.

To verify the ability of stable effector cells to fuse with stable target cells, we directly visualized the fusion events using a fluorescent protein as a reporter for fusion. This method allowed us to distinguish fusion events from any cell aggregates. For this purpose, we used target cells that were stably transfected with a plasmid expressing a green fluorescent protein (GFP) reporter protein under the control of a tTA-inducible promoter. Stable GFP-based Cf2Th-CD4/CCR5 target cells (Cf2-tetO-GFP) were cocultivated in the absence of Dox with either H-AD8 or H-JRFL effector cells, resulting in recognizable syncytia that were GFP-positive. H-JRFL cells were more fusogenic than H-AD8, as indicated by the number and extent of the fusion events. No syncytia could be detected when Dox was added to the medium. Notably, the pattern of fluorescence during fusion was quite different and more disperse than the pattern observed during transient expression of GFP in the target cells (Figure 3). This may be attributed to the ability of GFP to diffuse to all cells that form the syncytium, extending beyond the limits of a single cell.

### Effect of assay parameters on the fusion readout

**Figure 4:**
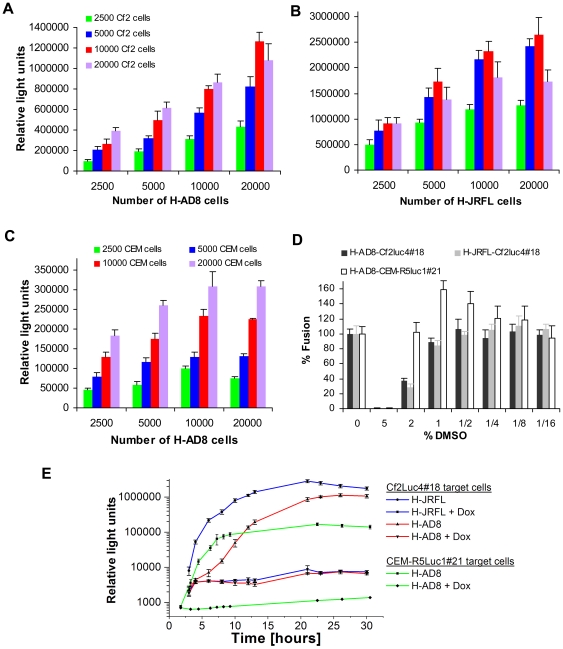
Effects of different assay parameters on the fusion readout. **A, B**. The influence of changes in the number of Cf2luc4#18 target cells (designated Cf2 cells), and either H-AD8 (**A**) or H-JRFL (**B**) effector cells, on the readout of the cell-cell fusion assay are shown. Color codes are identical for both panels. **C.** Same as (**A**) but with CEM-R5Luc1#21 target cells. **D.** The effect of different concentration of DMSO on the cell-cell fusion assay. **E.** Time course of fusion of H-AD8 and H-JRFL cells with Cf2Luc4#18 target cells and of fusion of H-AD8 cells with CEM-R5Luc1#21 cells. Results are representative from those obtained in three independent experiments.

The effects of varying the number of cells, time of incubation and DMSO concentration on the assay readout were evaluated with the different effector and target cell types. To study the effect of changes in the number and ratio of the cells, the number of the two target and two effector cells was varied in the fusion assay (Figure 4). Assay readout was robust over a range of target:effector cell ratios, and generally increased with higher numbers of both effector and target cells. For Cf2Luc4#18 target cells, the fusion readout with H-JRFL cells was generally higher and more readily saturated than that with H-AD8. Cell-cell fusion was also monitored over time with different combinations of effector and target cells (Figure 4E). Fusion of Cf2Luc4#18 target cells with H-JRFL cells showed rapid fusion kinetics and reached a higher maximum compared to fusion with H-AD8 cells. Fusion of CEM-R5Luc1#21 target cells with H-AD8 cells exhibited rapid kinetics and a very low background. In all cases, the assay readout was completely abolished by Dox, with only a slight increase in background during the time course.

The effect of DMSO (dimethyl sulfoxide), which is routinely used to dissolve chemical compounds, on the cell-cell fusion assay was also studied. For the fusion assay using Cf2Luc4#18 target cells, concentrations of up to 1% DMSO did not significantly change the assay readout, demonstrating a high tolerance of the system to this solvent and its suitability for use in large-scale screening. Interestingly, the fusion assay using CEM-R5Luc1#21 target cells tolerated DMSO concentrations as high as 2%, and moderate concentrations of DMSO resulted in higher assay readouts (Figure 4D).

### Levels of control on the cell-cell fusion system

**Figure 5:**
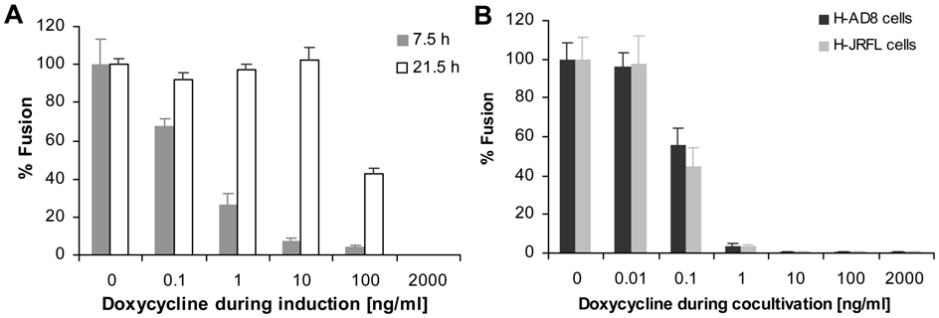
Two levels of control on the fusion assay. **A.** The levels of expression of HIV-1 Envs in the H-AD8 effector cells were controlled by varying the concentration of Dox during induction. Cells were washed prior to fusion, and allowed to fuse with Cf2Luc4#18 cells in Dox-free medium, initiating full induction of HIV-1 Envs. The fusion readout is shown after 7.5 and 21.5 hours. **B.** The level of expression of HIV-1 Envs in H-AD8 and H-JRFL cells, as well as that of F-Luc in the Cf2Luc4#18 target cells, was suppressed during fusion by adding Dox into the medium. The results shown are representative of those obtained three independent experiments.

The use of Dox enables two levels of control in the cell-cell fusion system. tTA activates the expression of HIV-1 Envs in effector cells, as well as the expression of F-Luc in target cells after productive cell-cell fusion. Thus, regulation of tTA activity by Dox enables two types of control over the fusion process: 1) manipulation of the level of Env expression in the effector cells by modifying Dox concentrations during induction; and 2) manipulation of the fusion readout by modifying Dox concentrations during cell-cell fusion. The first type of control was tested by inducing H-AD8 cells to express different levels of Envs by using decreasing concentrations of Dox; the cells were then washed to remove any Dox and incubated with Cf2Luc4#18 target cells in the absence of Dox (Figure 5). The extent of fusion after 7.5 hours correlated with the level of Env expression; i.e., high fusion readout was associated with low Dox concentrations. This effect was temporary and substantially decreased with a longer incubation time of 22.5 hours; such an outcome was expected because full expression of HIV-1 Envs occurred beginning at the time of cocultivation of effector cells and target cells, regardless of the induction condition. The second level of control involved modification of the fusion readout when Dox was added during the fusion process. For both types of effector cells, addition of Dox during the cocultivation of effector and target cells decreased the fusion readout in a dose-dependent manner, with almost complete suppression at 1 ng/ml. Accordingly, addition of 2 µg/ml Dox, which completely abolished tTA-related F-Luc expression, was used in our experimental system to define the background level of cell-cell fusion.

### Dual reporter system

**Figure 6:**
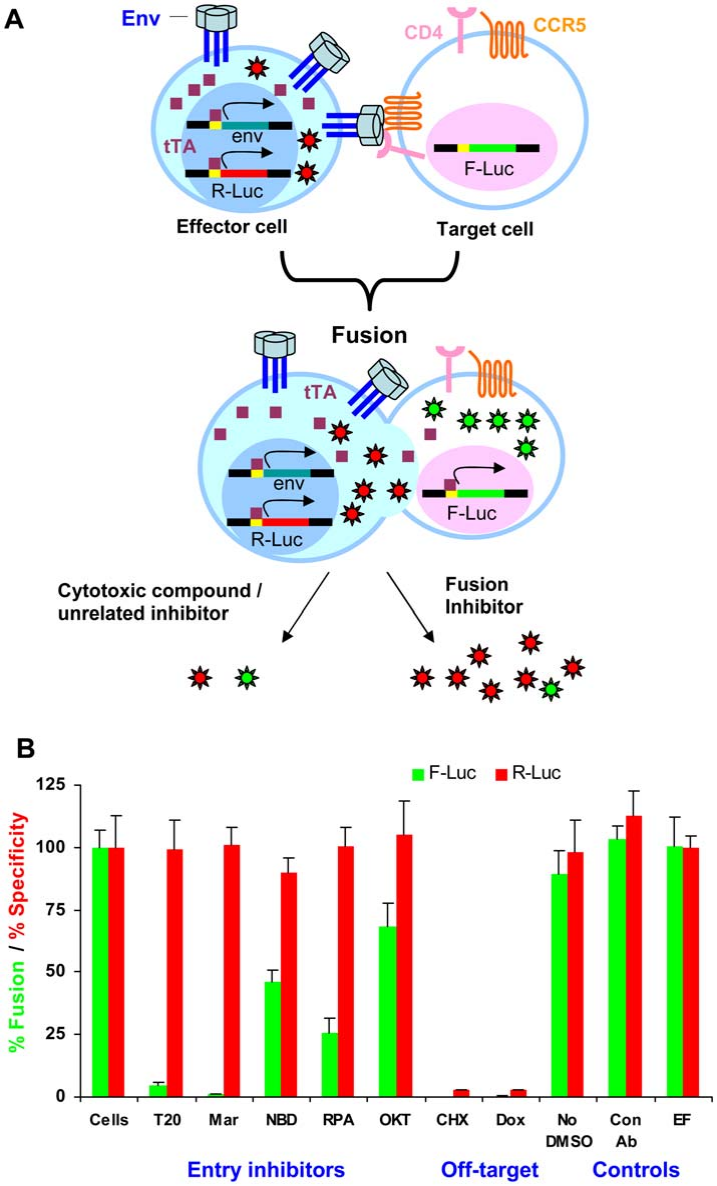
Design and testing of a dual reporter assay measuring inhibition efficiency and specificity. **A.** A dual reporter system was designed by incorporating the R-Luc gene, under the control of a tTA-responsive promoter, into effector cells. When these cells are fused to target cells, two readouts are measured sequentially: F-Luc activity measures the extent of cell-cell fusion and R-Luc activity measures any off-target effect. Specific fusion inhibitors are expected to give low F-Luc and high R-Luc activities, whereas unrelated inhibition results in low activities of both luciferases. **B.** Various compounds were tested with the dual reporter system using H-AD8#15Ren effector cells and CEM-R5L1#21 target cells. Both F-Luc and R-Luc activities were measured and normalized to those activities seen in cells without any inhibitor. The final concentration of DMSO during the cell-cell fusion assay was 0.1% unless otherwise indicated. T20, T20 peptide (118 nM); Mar, Maraviroc (1.18 µM); CHX, cycloheximide (100 µg/ml); RPA, RPA-T4 (anti-CD4 antibody, 1 nM); OKT, OKT4 (anti-CD4 antibody, 1 nM); Con.Ab, Control antibody (1 nM); NBD, NBD-556 (9.1 µM); Dox, doxycycline (2 µg/ml); EF, efavirenz (an HIV-1 reverse transcriptase inhibitor, 312 nM).

A potential problem in using the cell-cell fusion assay for large-scale screening is that the final readout could be decreased by compounds that have non-specific effects on cell viability, or by compounds that specifically inhibit the tTA-based induction system. Such unrelated effects can be mistakenly attributed to Env-specific inhibition, which also decreases the assay readout. To recognize such false positives, we introduced a tTA-regulated gene encoding a second reporter, Renilla luciferase (R-Luc), into H-AD8 effector cells (generating H-AD8#15Ren cells, Figure 6A). In this setting, induction of the Envs by removal of Dox also induces the expression of R-Luc in the effector cells. Following effector-target cell cocultivation, both luciferase activities can be measured sequentially within the same cell lysate. F-Luc activity measures the extent of cell-cell fusion, whereas R-Luc activity evaluates off-target effects. To assess the ability of this system to distinguish specific fusion inhibitors from unrelated or cytotoxic ones, we tested different compounds. All four HIV-1 entry inhibitors (T20, Maraviroc, RPA and NBD-556) exhibited specific inhibition with low fusion (F-Luc) and high specificity (R-Luc) signals when compared to a control fusion assay without inhibitors (Figure 6B). The efficient entry inhibitors T20 and Maraviroc were very potent and led to almost complete inhibition without any substantial off-target effects. The weak entry inhibitor NBD-556 was less effective, and inhibited approximately 50% of the fusion activity at a concentration of 9.1 µM. Low inhibition by the OKT4 anti-CD4 antibody was observed with CEM-R5Luc1#21 target cells but not with Cf2Luc4#18 target cells. Low inhibition of HIV-1 Env-mediated cell-cell fusion has been previously observed for the highest OKT4 concentrations used in a different assay system [61]. Importantly, specific inhibition was documented for inhibitors that are directed against different components of the HIV-1 entry process, including gp41 (T20), gp120 (NBD-556), CCR5 (Maraviroc) and CD4 (RPA). In contrast, the cytotoxic translation blocker cycloheximide and Dox, which controls the expression of both luciferases, decreased both F-Luc and R-Luc signals. A control antibody and Efavirenz, as expected, did not have any effect on the activity of either luciferase. Thus, the assay is capable of distinguishing a specific inhibitor of HIV-1 Env-mediated membrane fusion from off-target inhibitors.

**Figure 7:**
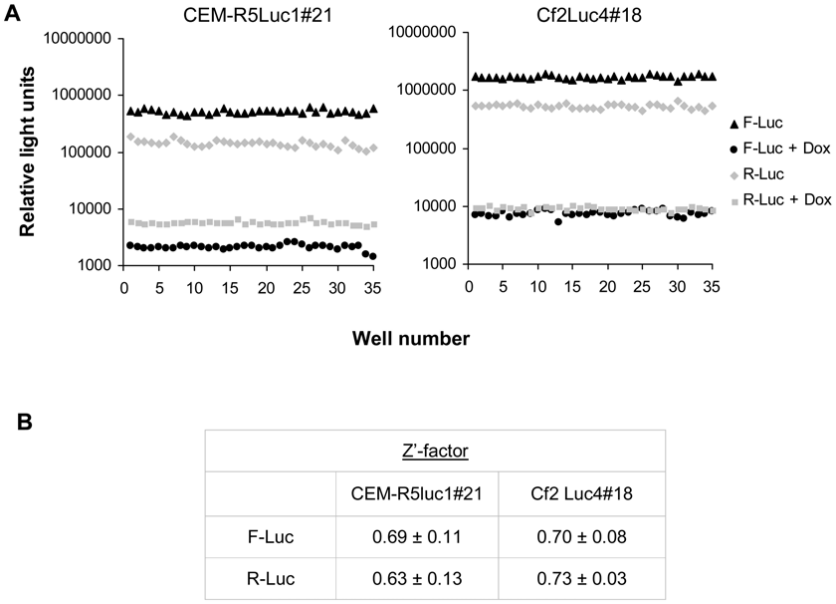
Statistical analysis of the dual reporter system with two different target cells. **A.** Distribution of F-Luc and R-Luc signals obtained in the cell-cell fusion assay conducted with or without 2 µg/ml Dox. Readouts of fusion without Dox are from 35 wells of a single plate and those with Dox are from 35 wells from multiple plates. **B.** Statistical analysis for the precision of the system. Data from 5 (CEM-R5Luc1 cells) or 4 (Cf2Luc4#18) independent experiments were used to calculate the Z'-factor of the assay for each luciferase. Means ± standard deviations are shown for each cell type.

Statistical analysis showed that the fusion readout as well as the expression of R-Luc were reproducible in the dual reporter system (Figure 7). Low variation was observed for readouts from replicate wells, and incubation with Dox consistently and thoroughly suppressed both luciferase activities. The calculated Z'-factor was high for both target cells, demonstrating their suitability for large-scale screening. A slightly higher value with lower standard deviation was observed for Cf2Luc4#18 target cells compared with the values for CEM-R5Luc1#21 cells.

### Validation of the system with known inhibitors

**Figure 8:**
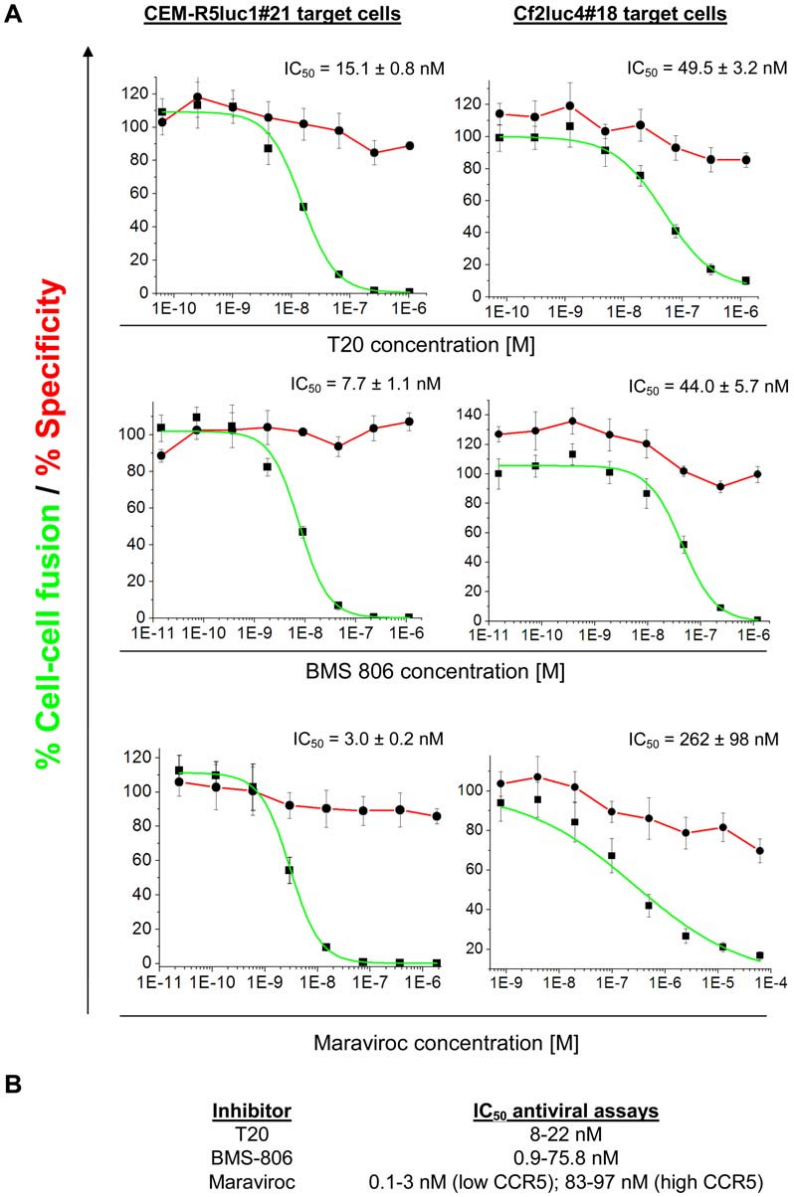
Dose-response curves for inhibition efficiency and specificity of known HIV-1 entry inhibitors. **A.** The gp41-directed T20 peptide, gp120-directed BMS-806, and CCR5-directed Maraviroc were tested for inhibition and off-target effects with the dual reporter system using two different target cells, CEM-R5Luc1#21 (left) and Cf2Luc4#18 (right). Inhibition data were fitted to the logistic equation and the IC_50_ value for each tested compound and each target cell type was calculated. R^2^>0.96 for all curves. Red lines display relative R-luc activity and indicate the specificity of a compound; green lines are fitted curves for relative F-luc activity and indicate the inhibition of cell-cell fusion for each compound. The results shown are representative of those obtained in three or four independent experiments, each performed with more than three replicates. **B.** Reported IC_50_ values for the three inhibitors in antiviral assays [24], [27], [37], [62], [71], [72] (inhibition of the HIV-1_MN_ isolate by BMS-806 was reported to be 743 nM [24] and this atypical value is excluded from the table).

The ability of the cell-cell fusion system to monitor inhibition efficiency as well as specificity can be utilized to measure the inhibitory and cytotoxic concentrations of a specific inhibitor, enabling assessment of the therapeutic index. Moreover, such measurements can be used to evaluate the contribution of any cytotoxic effects to compound inhibition at any given concentration. To evaluate this capability, the dose-response profiles of three known inhibitors were studied, using both target cells (Figure 8). The CEM-R5Luc#21 cells were more sensitive to inhibition by all three inhibitors and the IC_50_ values closely matched published antiviral data (Figure 8B). Inhibition of cell-cell fusion by Maraviroc was tightly related to the level of CCR5 expression on the target cells, as previously published [62]. For fusion with the low-CCR5-expressing CEM-R5Luc1#21 target cells, the calculated IC_50_ was about 3 nM, whereas this value increased about 100-fold for the high-CCR5-expressing Cf2Luc4#18 cells. Less profound differences between the assay results obtained with different target cells were observed for inhibition by T20 and BMS-806. Inhibition of fusion with CEM-R5Luc1#21 target cells was about 3-fold more efficient for T20 and about 6-fold more efficient for BMS-806, compared to the inhibition of fusion with Cf2Luc4#18 cells. The R-Luc inhibitory activity of each compound was also evaluated for all tested concentrations and was used to generate a “cytotoxic” curve (Figure 8). High concentrations of T20 and Maraviroc appeared to be slightly toxic in the assay in which HeLa cells and Cf2Luc4#18 cells were cocultivated, as indicated by the small decrease in R-Luc activity. Except for this effect, none of the compounds was significantly cytotoxic and no substantial decrease in R-Luc activity was observed at any tested concentration. These results confirm the high therapeutic indices of T20, Maraviroc and BMS-806.

## Discussion

We have developed an advanced cell-cell fusion system with a unique configuration in which the tTA transactivator was exploited to regulate expression of all components including HIV-1 Envs, F-Luc and R-Luc. Cocultivation of Env-expressing effector cells and receptor-expressing target cells resulted in robust and reproducible cell-cell fusion. The dual system effectively distinguished specific fusion inhibitors from non-related ones, and reliably measured inhibitory concentrations as well as cytotoxicity for known fusion inhibitors.

The tetracycline–inducible expression system was selected because it is well characterized and offers very tight regulation, with low background levels of transcription and high expression levels [60]. Interestingly, Env expression from the non-lentiviral vector pGL4.2-JRFL was consistently higher than Env expression from the lentiviral vector plvx-JRFL in both transiently and stably transfected cells. Although the tTA promoter and Env sequences are identical in both vectors, the flanking sequences in the vector may suppress or enhance tTA-mediated transcription; moreover, distinct integration sites in the stable cell lines may exert different effects on transcription.

The specific design of our cell-cell fusion system offers several advantages: 1) the use of inducible stable cell lines avoids Env-related toxicity and provides consistent expression of HIV-1 Envs relative to repeated transient transfections, which, in addition to being more variable, are time- and resource-consuming; 2) HIV-1 Env expression is tightly regulated and derived from a codon-optimized gene, alleviating the requirement for the HIV-1 Rev protein in the system; 3) tight control of tTA allows a complete shutoff of transcription of all components of the system, as well as the ability to control Env expression level prior to the cocultivation of effector and target cells; 4) R-Luc activity can be used as a surrogate for the level of Env expression on the effector cells because both Envs and R-Luc are regulated by the same tTA protein in these cells; 5) The system can distinguish tTA inhibitors, which will result in off-target effects, from specific fusion inhibitors because R-Luc activity is regulated by tTA; and 6) dual measurements of luciferase activity allow the evaluation of both efficiency and specificity of inhibition in the same cells under the identical experimental conditions, avoiding potential errors associated with measuring these two properties separately. The only limitation of our system is the inability to distinguish fusion inhibitors from specific F-Luc inhibitors. F-Luc inhibitors will lower F-Luc activity without a significant decrease in R-Luc activity, and therefore will be scored as specific fusion inhibitors (false positives). Nevertheless, such compounds are presumably very rare, are easily identifiable in secondary assays, and thus should not significantly compromise the utility of the system to measure specificity.

Some of the above features of our system have been previously used in transient or stable fusion assays. However, these systems have not incorporated all of these features into a single complete system; moreover, the manner in which these elements were utilized differs from ours. One assay monitored the fusion of transiently transfected 293T cells [45]; in this case, both transfection efficiency and cytotoxicity were measured with a single R-Luc marker, and thus the contribution of each to the total effect was unknown. A tTA-based system has been used for transient expression of Envs and for detection of fusion inhibition [24]. Another fusion system with inducible expression of HIV-1 Envs has also been reported [57]. In this system, Dox was used for induction and was required throughout the fusion assay. Coexpression of HIV-1 Rev was required for Env expression, and HIV-1 Tat was used as a transactivator to trigger F-Luc expression in the target cells. Identifying fusion inhibitors in this setting requires an additional step to confirm that the compounds do not inhibit either HIV-1 Rev or Tat. In contrast to our system, this earlier assay did not contain a built-in ability to measure off-target effects.

Our cell-cell fusion system, like other fusion systems, is focused on a single step in the HIV-1 life cycle. Such a strategy will not identify inhibitors of other steps in the virus life cycle, but has several advantages. Compared with more comprehensive antiviral assays, the more focused cell-cell fusion assay requires a shorter time of incubation, may be less sensitive to non-specific effects, and allows the target of any inhibitor to be easily identified. In addition, the cell-cell fusion system does not require the use of special biosafety containment facilities, as no infectious virus is used.

Our dual reporter system was successfully tested against a wide range of inhibitors and control molecules. The system detected known fusion inhibitors and underscored their specificity, whereas control agents did not significantly affect cell-cell fusion. Specific inhibition was documented for inhibitors that target different Env or receptor components of the HIV-1 entry process; for different types of inhibitory molecules such as chemical compounds, peptides and antibodies; and for inhibitors with diverse levels of efficiency [ranging from an IC_50_ of ∼10 µM (NBD-556) to ∼3nM (Maraviroc)]. Non-related effects of cycloheximide and tTA-inhibitors were clearly categorized as off-target, as they completely abolished the activity of both luciferases. Measurements of inhibitory concentrations and cytotoxicity of known inhibitors validated the utility of the fusion system. The system was efficiently inhibited in a dose-response manner by all known inhibitors, with different efficacy for the two target cells types tested. CCR5-directed Maraviroc was the most potent inhibitor of fusion to CEM-R5Luc1#21 cells and a less efficient inhibitor for fusion to Cf2Luc4#18 cells. Such differences in potency are expected, since the lower number of target CCR5 molecules on CEM cells, relative to those on Cf2Th cells, enables effective inhibition by even low concentrations of Maraviroc. Our results suggest that the distinct properties of the two target cells available for this cell-cell fusion system may exhibit different levels of sensitivity for the detection of particular types of inhibitors. CEM-R5Luc1#21 cells grow in suspension and are derived from human T-cells that are closely related to authentic target cells during HIV-1 infection; fusion with CEM-R5Luc1#21 cells is inhibited very efficiently by known fusion inhibitors. Cf2Luc4#18 are adherent cells, and are less sensitive to fusion inhibitors, but exhibited a slightly higher Z'-factor with less variation.

Adaptation of the dual reporter system to high-throughput screening in a 384-well format may require additional optimization of cell numbers to adjust for the lower volumes. In addition, commercial luciferase substrate solutions, such as Steady-Glo (Promega), which are designed to have a long half-life for batch processing, have lower luminescence intensities in comparison to other formulations that are designed for high sensitivities (Steady-Glo Technical Manual, Promega). Using such a substrate resulted in lower readouts than the luciferase substrate that was used in our study (data not shown). Moreover, because some decrease of signal readout was observed after long-term propagation of the effector cells, the use of low passage cells is recommended. Preliminary data from scaled-up pilot studies indicate that the cell-cell fusion assay is robust and underscore the importance of using a control system for excluding off-target compounds.

In summary, our assay is simple and efficient. The system has high dynamic range, tolerates DMSO, is precise (high Z'-factor scores) and has been validated with a panel of reference inhibitors directed against different steps in the fusion process, yielding inhibitory concentrations consistent with published data. The dual reporter system can be used for detailed characterization of inhibition versus cytotoxic properties of entry inhibitors. In addition, the system is suitable for a high-throughput screen of chemical compounds and adds a unique layer to the already existing cell-cell fusion systems. We are currently developing additional cell-cell fusion assays that utilize Env derived from different primary HIV-1 isolates. Combining the screening with secondary assays that utilize cells expressing Env from different HIV-1 isolates may identify broad-range fusion inhibitors that can generate novel lead compounds for further development as therapeutics or prophylactic microbicides.

## Materials and Methods

### Reagents and antibodies

Maraviroc and T20 were obtained from the AIDS Research and Reference Reagent Program, Division of AIDS, NIAID, NIH. Cycloheximide was from Sigma. RPA-T4, 2D7 and control isotype antibodies were purchased from BD Biosciences; OKT4 was obtained from eBiosciences.

### Cell lines

HeLa-TetOff cells stably expressing the tetracycline-controlled transactivator were purchased from Clontech, and maintained in DMEM supplemented with 10% heat-inactivated fetal calf serum (FCS), 2mM L-glutamine, 100 units/ml penicillin, 100 µg/ml streptomycin, and 100 µg/ml geneticin (all from Gibco). The canine Cf2Th-CD4/CCR5 cell line stably expressing the human CD4 receptor and CCR5 coreceptor [63] was maintained in the same medium supplemented with 400 µg/ml geneticin and 200 µg/ml hygromycin (Gibco). Human embryonic kidney 293T/17 cells (designated 293T) were obtained from the American Type Culture Collection (ATCC) and maintained in the same medium but without any selection antibiotics. CEM.NKR-CCR5 cells were obtained through the AIDS Research and Reference Reagent Program, Division of AIDS, NIAID, NIH from Dr. Alexandra Trkola [64], [65], [66], and were maintained in RPMI-1640 supplemented with 10% FCS, 2mM L-glutamine, 100 units/ml penicillin, and 100 µg/ml streptomycin.

### Plasmid construction

The plvx-AD8 plasmid was generated by PCR amplification of the HIV-1_AD8_ *env* gene from pcc-AD8on (a gift from Dr Xinzhen Yang, Beth Israel Deaconess Medical Center) and cloning the PCR product into the BamHI/EcoRI restriction sites of the plvx-Tight-Puro vector (Clontech). The HIV-1_AD8_ *env* sequences encoding the first 610 residues of Env are codon-optimized and enable Env expression without the need for HIV-1 Rev. The plvx-JRFL plasmid was generated in a similar manner, by cloning the fully codon-optimized version of HIV-1_JRFL_ *env* into plvx-Tight-Puro. The pGL4.22-JRFL plasmid was generated by digesting plvx-JRFL with XhoI/EcoRI and subcloning the ∼2900 bp fragment (containing the tetracycline-controlled promoter and HIV-1 *env* sequences) into the same restriction sites in pGL4.22[luc2CP_Puro] (Promega). The pGL4.78_tetO_Renilla plasmid was generated by PCR amplification of the tetracycline-controlled promoter and cloning it into NheI/HindIII restriction sites in pGL4.78[hRlucCP_Hygro] (Promega). The plvx-tetO-GFP plasmid was generated by cloning the GFP gene into plvx-Tight-Puro-Luc (Promega), replacing the luciferase gene with the GFP gene. All plasmids were sequenced to verify the correct sequence of the inserts.

### Generation of stable cell lines

Stable gene transfer of the AD8 *env* gene was mediated by pseudoviruses that were prepared by transfecting 293T cells with plvx-AD8, and a mix of helper virus expression plasmids, according to the manufacturer's instructions (Clontech). Pseudoviruses were used to infect HeLa-TetOff cells that were then selected with 1 µg/ml puromycin to generate H-AD8 cells. H-JRFL cells were generated by transfecting the HeLa-TetOff cells with pGL4.2-JRFL, followed by selection with 1 µg/ml puromycin. From the first day after infection (or transfection), 2 µg/ml Dox (Clontech) was added to the growth medium to avoid any detrimental effect caused by the expression of the HIV-1 Envs. More than 20 different single clones were isolated after propagating H-AD8 cells in 96-well plates under limiting dilution conditions, and each clone was tested for its fusion activity. H-AD8#15Ren was generated by transfecting AD8 clone #15, which demonstrated the most efficient fusion to target cells in pilot assays, with the pGL4.78_tetO_Renilla plasmid and subsequent selection with 200 µg/ml hygromycin.

Cf2luc4 and Cf2-tetO-GFP target cells were prepared in a manner similar to that described above, using either plvx-Tight-Puro-Luc plasmid (for Cf2luc4 cells, Clontech) or plvx-tetO-GFP (for Cf2-tetO-GFP cells) to prepare pseudoviruses. Viruses were used to infect Cf2Th-CD4/CCR5 cells that were subsequently selected with 4 µg/ml puromycin. CEM-R5Luc1 target cells were generated by infecting CEM.NKR-CCR5 cells with pseudoviruses that were prepared with the plvx-Tight-Puro-Luc plasmid and subsequent selection with 1 µg/ml puromycin. Single clones were isolated as described above for H-AD8 clones. Cf2Luc4#18 and CEM-R5Luc1#21 exhibited high fusion activity and the lowest background in the cell-cell fusion assay.

### Flow cytometry

Half a million cells were analyzed by flow cytometry as previously described [67], but with primary antibody incubation for 30 min, and secondary antibody (R-Phycoerythrin-conjugated goat anti-mouse IgG, Invitrogen) incubation for 10 min, both at room temperature. Cells were analyzed with a BD FACSCanto II flow cytometer (BD Biosciences) and results were displayed using Flow Jo software (Tree Star).

### Cell-cell fusion assay

Effector cells were washed 3 times with PBS, trypsinized, and 1×10^6^ cells were seeded at a concentration of 1×10^5^/ml in DMEM medium without phenol red, containing Tetracycline-approved FBS (Clontech) and supplemented with 100 µg/ml geneticin and 1 µg/ml puromycin (200 µg/ml hygromycin was also added for H-AD8#15Ren cells). After incubating for 3 hours at 37°C, the medium was replaced by fresh medium to remove any traces of Dox and to enable full induction of HIV-1 Env expression. Cf2Luc4#18 target cells were seeded at 1×10^5^/ml in the same medium as above, but supplemented with 400 µg/ml geneticin, 4 µg/ml puromycin and 200 µg/ml hygromycin. After overnight incubation (∼24 hours), effector and target cells were detached with 5 mM EDTA in PBS or with a minimal amount of trypsin, washed once with PBS and then added sequentially to a 96-well B&W isoplate (PerkinElmer) in the above medium without any selection antibiotics. After an overnight incubation at 37°C, the medium was aspirated, 30 µl of passive lysis buffer (Promega) was added to each well, and F-Luc activity was measured with a Centro LB 960 luminometer (Berthold Technologies, TN, USA). One hundred microliters of assay buffer (15 mM MgSO_4_, 15 mM KH_2_PO_4_/K_2_HPO_4_ pH 7.8, 1 mM ATP and 1 mM DTT) was injected, followed by a 50 µl injection of 1 mM luciferine (BD Biosciences); luminescence was measured for 10 sec. For dual assay measurements, this procedure was followed by injecting 50 µl of quench-substrate solution [2.2 M NaCl, 4.4 mM EDTA pH 8, 0.44 M KH_2_PO_4_/K_2_HPO_4_ pH 5.1, 0.88 mg/ml BSA, 2.6 mM sodium azide and 2.9 µM coelenterazine (Promega)] and measuring R-Luc activity for 10 sec [68]. When CEM-R5luc1#21 target cells were used for fusion, they were spun down and resuspended in RPMI-1640 medium containing Tetracycline-approved FBS (Clontech) without Phenol Red prior to the assay. Approximately 1×10^4^ target cells were added to each well containing the same number of pre-incubated effector cells, and the fusion assay was carried out in either DMEM/RPMI-1640 (50%/50%) or complete RPMI-1640 medium. For the kinetic experiments, cells were detached with 5 mM EDTA in PBS, lysed after the specified time of target-effector cell cocultivation, and frozen at −80°C until luciferase activity was measured. For inhibition experiments, cells and inhibitors were sequentially added to the wells, and the cell-cell fusion assay was carried out for 13-15 hours at 37°C. Inhibition data were fitted to the logistic equation using the nonlinear curve fit module in Origin 8.1 software, as previously described [69]. For statistical analysis, the Z'-factor was calculated using the fusion readout as the high signal and fusion in the presence of Dox as the low signal, as described [70]: Z'  =  1 − [(3*SD_fusion_ + 3*SD_fusion+Dox_)/(Mean_fusion_ − Mean_fusion+Dox_)], where SD represents the corresponding standard deviation of fusion with or without Dox.

### Immunoprecipitation assay

Approximately 2–3 ×10^5^ effector cells (H-JRFL or clone#7 of H-AD8) were induced for 48 hours at 37°C, metabolically labeled overnight with 30 µCi ^35^S and then lysed with RIPA buffer (150 mM NaCl, 1.0% IGEPAL CA-630, 0.5% sodium deoxycholate, 0.1% SDS, 50 mM Tris, pH 8.0) that contained protease inhibitor cocktail (Roche). The lysate was cleared and subjected to immunoprecipitation with serum from HIV-1-infected individuals followed by SDS-PAGE and autoradiography.

### Fluorescence microscopy

Effector cells were incubated with Cf2-tetO-GFP target cells, as described in the fusion assay section above, in 96-well Optilux black plates with clear bottoms (BD Biosciences). After an approximately 40-hour incubation at 37°C, cells were visualized under a Nikon Eclipse Ti-S fluorescence microscope equipped with a DS-Qi1 camera, and images of live cells were acquired.

## Supporting Information

Figure S1 **Transient expression levels and fusion activity of HIV-1 Envs expressed by different vectors. A.** Expression of HIV-1 Envs using three different vectors was measured in HeLa-TetOff cells. Two lentivirus vectors (plvx-AD8 and plvx-JRFL) were used for induced expression of either HIV-1_AD8_ or HIV-1_JR-FL_ Envs, respectively, and one nonlentiviral vector (pGL4.2-JRFL) for induced expression of HIV-1_JR-FL_ Envs. HeLa-TetOff Cells were transiently transfected in the presence of varying concentrations of Dox or without any Dox; cells were lysed and analyzed by Western blotting (as described in Method S1). Lane 1, untransfected cells; lane 2, cells transfected with a control vector (plvx-Tight-Puro); lanes 3-7, cells transfected with 0.8 µg of the indicated plasmid in the presence of specified concentrations of Dox; lane 8, recombinant HIV-1_YU2_ gp120 (positive control). **B.** Effector cells were transiently cotransfected with the specified plasmid and an R-Luc-based vector for normalization (as described in Method S1). Cf2Th-CD4/CCR5 cells were transfected with plvx-Tight-Puro-Luc. The cells were allowed to fuse and F-Luc activity was measured. Fusion was normalized for transfection efficiency by the R-Luc activity.
(TIF)

Methods S1 (DOC)
