# Supplementary figures and images for: An Inducible Cell-Cell Fusion System with Integrated Ability to Measure the Efficiency and Specificity of HIV-1 Entry Inhibitors
Source: PLoS One. 2011 Nov 1;6(11):e26731. doi: 10.1371/journal.pone.0026731 (PMC3206054; doi:10.1371/journal.pone.0026731)

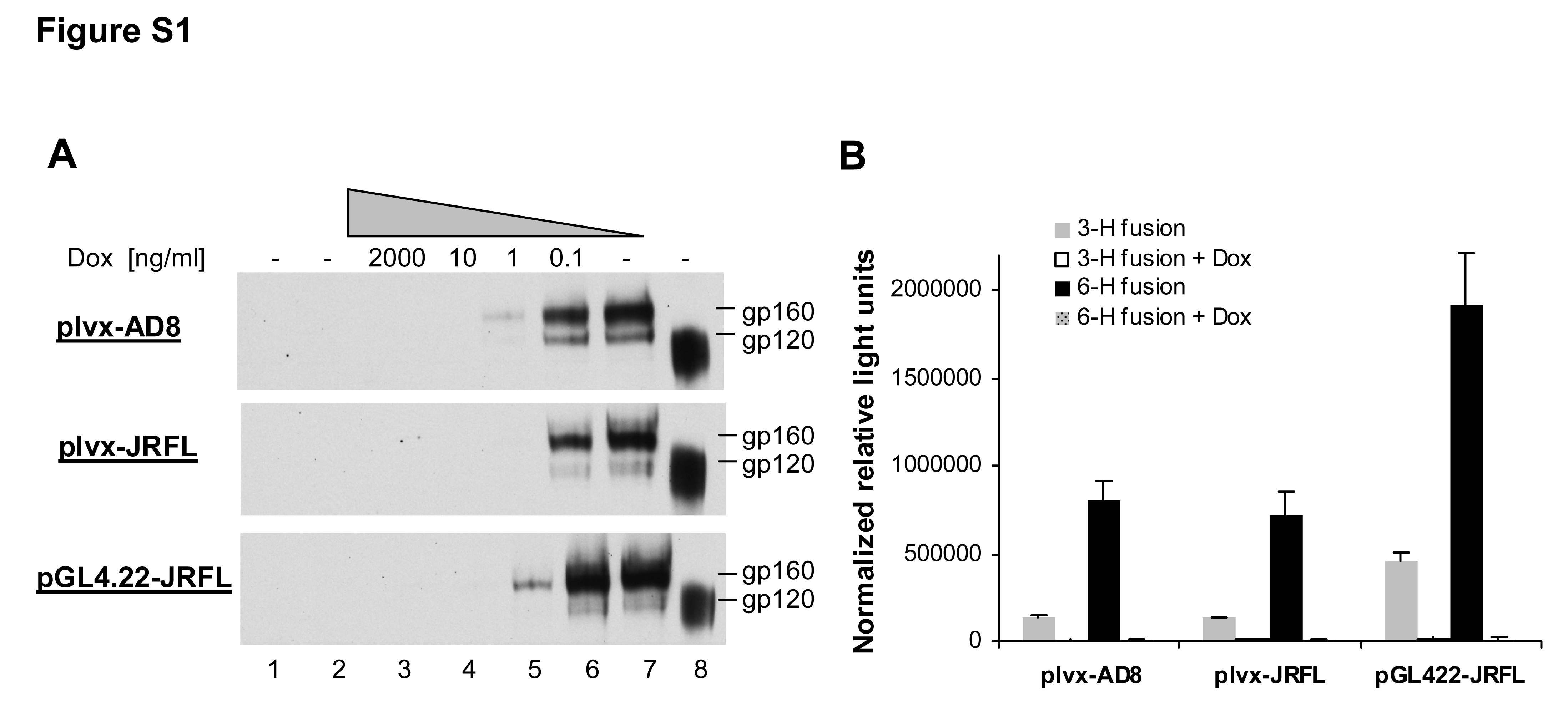

Supplement: Figure S1 — Transient expression levels and fusion activity of HIV-1 Envs expressed by different vectors. A. Expression of HIV-1 Envs using three different vectors was measured in HeLa-TetOff cells. Two lentivirus vectors (plvx-AD8 and plvx-JRFL) were used for induced expression of either HIV-1AD8 or HIV-1JR-FL Envs, respectively, and one nonlentiviral vector (pGL4.2-JRFL) for induced expression of HIV-1JR-FL Envs. HeLa-TetOff Cells were transiently transfected in the presence of varying concentrations of Dox or without any Dox; cells were lysed and analyzed by Western blotting (as described in Method S1). Lane 1, untransfected cells; lane 2, cells transfected with a control vector (plvx-Tight-Puro); lanes 3-7, cells transfected with 0.8 µg of the indicated plasmid in the presence of specified concentrations of Dox; lane 8, recombinant HIV-1YU2 gp120 (positive control). B. Effector cells were transiently cotransfected with the specified plasmid and an R-Luc-based vector for normalization (as described in Method S1). Cf2Th-CD4/CCR5 cells were transfected with plvx-Tight-Puro-Luc. The cells were allowed to fuse and F-Luc activity was measured. Fusion was normalized for transfection efficiency by the R-Luc activity. (TIF) [file pone.0026731.s001.tif]
