# Supplementary material for: An Inducible Cell-Cell Fusion System with Integrated Ability to Measure the Efficiency and Specificity of HIV-1 Entry Inhibitors
Source: PLoS One. 2011 Nov 1;6(11):e26731. doi: 10.1371/journal.pone.0026731 (PMC3206054; doi:10.1371/journal.pone.0026731)
Supplement: Methods S1 — (DOC) [file pone.0026731.s002.doc]

**METHOD S1**

**Transient transfection.**  Approximately 1 x 10^6^ HeLa-TetOff cells in DMEM that contained Tetracycline-approved FBS were transfected in a 10-cm plate with 0.8 µg of either plvx-AD8, plvx-JRFL or pGL4.22-JRFL plasmids. For experiments in which transfection efficiency was normalized with R-Luc, 0.6 µg of each plasmid was cotransfected with 0.1 µg of pGL4.78_tetO_Renilla. Cf2Th-CD4/CCR5 cells were transfected in similar manner, with 0.8 µg of plvx-Tight-Puro-Luc. All cells were transfected using Effectene (Qiagen) according to the manufacturer’s instructions. For cell-cell fusion assays of transiently transfected cells, 1 x 10^4^ effector cells and 5 x 10^3^  target cells were used and the fusion readout was further normalized for transfection efficiency by R-Luc activity.

**Western blotting.** Transfected HeLa-TetOff cells were incubated at 37°C for 72 hours, washed with PBS and lysed for 30-60 min at 4°C with RIPA buffer that contained protease inhibitor cocktail (Roche). Lysates were cleared by centrifugation at 20,000 xg at 4°C for 10 min and the protein concentration in the supernatant was measured with a BCA protein assay kit (Pierce). Equal amounts of protein from each sample were separated on 8-16% Tris-Glycine gels (Invitrogen) and transferred to a nitrocellulose membrane. After blocking with 5% dry skim milk in PBS, proteins were detected with a combination of serum from an HIV-1-infected individual (1:8000 dilution) and rabbit anti-gp120 polyclonal antibodies (1:200–1:1000 dilution, ImmunoDiagnostics), followed by a combination of horseradish peroxidase-conjugated goat anti-rabbit IgG (1:14000 dilution) and horseradish peroxidase-conjugated donkey anti-human IgG (1:20000 dilution), both from Jackson Immunolaboratories.
